# One in five, not one in 17, youth patients deteriorate during psychotherapy for depression

**DOI:** 10.1007/s00787-022-01943-6

**Published:** 2022-01-17

**Authors:** Andrew A. McAleavey

**Affiliations:** 1 Center for Health Research, Helse Forde, 45, Tefrevegen, 6812 Forde, Norway; 2 Department of Psychiatry, Weill Cornell Medical College, New York, USA

I read the recent article “The effects of psychological treatments of depression in children and adolescents on response, reliable change, and deterioration: a systematic review and meta‐analysis” by Cuijpers and colleagues [1] with great interest. The authors conducted a comprehensive meta-analysis and found that while psychotherapies for youth were more effective in treating depression than control conditions, a considerable portion of patients do not respond well in treatment. This is, overall, an important and extremely well-conducted study. Since there is no clear marker of depression outcome that is unequivocal and universal across all patients, the authors used several metrics to examine differences, including a common definition of response (50% reduction in symptoms), number needed to treat, relative risk, and an estimated percent of individuals reporting a difference at least as large as the reliable change index (RCI; [4]).

I was surprised, however, to see a common misinterpretation of the RCI, namely that it identifies individuals with “clinically significant deterioration” (p. 8). The RCI on its own is merely a critical value, on which observed change scores can be judged as “reliably” different from 0. The RCI provides little information about the clinical significance of changes: it tells us that an observed difference score is less than 5% likely to occur if the true score difference was exactly 0, but nothing specific about the size of the underlying true difference score. The true difference may be extremely small and not clinically meaningful even if the observed difference exceeds the RCI, and many people with substantial true difference scores will have observed difference scores less than the RCI. It only pertains to clinical significance when it is paired with some clinical criterion, which was the original suggestion of Jacobson and Truax [4] and Jacobson, Follette, and Revenstorf [3] when they developed the RCI.

In other words, the RCI identifies individuals who look like they probably had true difference scores different than 0, while the true difference score is how much change actually occurred in the construct of interest. Though we cannot identify true scores precisely for individuals, we can infer the distribution of true scores directly using the authors’ report, and therefore can compute how many people truly improved or deteriorated, not just how many people appear likely to have changed.

The authors would be able to do this directly with their data (see [5]), but we can also compute the true change score distribution solely from the RCI estimates they provided. To demonstrate: the authors report that 54% of patients in psychotherapy had reliable improvement, and 6% had reliable deterioration (for simplicity, I only use their point estimates). This is the same as saying that 54% of their sample had observed difference scores less than − 1.96 * S_diff_ and 94% had observed differences less than 1.96 * S_diff_, where S_diff_ is the standard error of the difference score, or equivalently, the standard deviation of measurement error in difference scores. If we assume that observed difference scores are drawn from a Gaussian distribution (the authors’ assumption), we can identify the observed difference score distribution has Mean = − 2.23 and SD = 2.70, in units of S_diff_. If the observed difference scores were available, we could simply calculate this directly, but solving simultaneous equations is straightforward enough.

Because the observed scores are the sum of the true and error scores, the underlying true difference score distribution is known as well. The true difference score distribution has the same mean as the observed scores, under normal assumptions. However, the observed distribution’s SD is inflated by error. Deriving the true score variance, however, is simple. Given this basic tenet of classical test theory:

$${\sigma }_{observed}^{2}={\sigma }_{true}^{2} + {\sigma }_{error}^{2}$$

it follows that:

$${\sigma }_{true}^{2}={\sigma }_{observed}^{2} - {\sigma }_{error}^{2}$$

and, therefore:

$${\sigma }_{true}=\sqrt{{\sigma }_{observed}^{2} - {\sigma }_{error}^{2}}$$

Since the authors defined the magnitude of the error component (${\sigma }_{error}$ = S_diff_), and we have an estimate of ${\sigma }_{observed}$ = 2.70, we can easily solve for ${\sigma }_{true}$. To interpret the values at ± 1.96, we will scale the entire equation by S_diff_, which means we can substitute ${\sigma }_{error}$ = S_diff_ = 1. Therefore,

$${\sigma }_{true}=\sqrt{{2.70}^{2} - {1}^{2}}$$

**Fig. 1:**
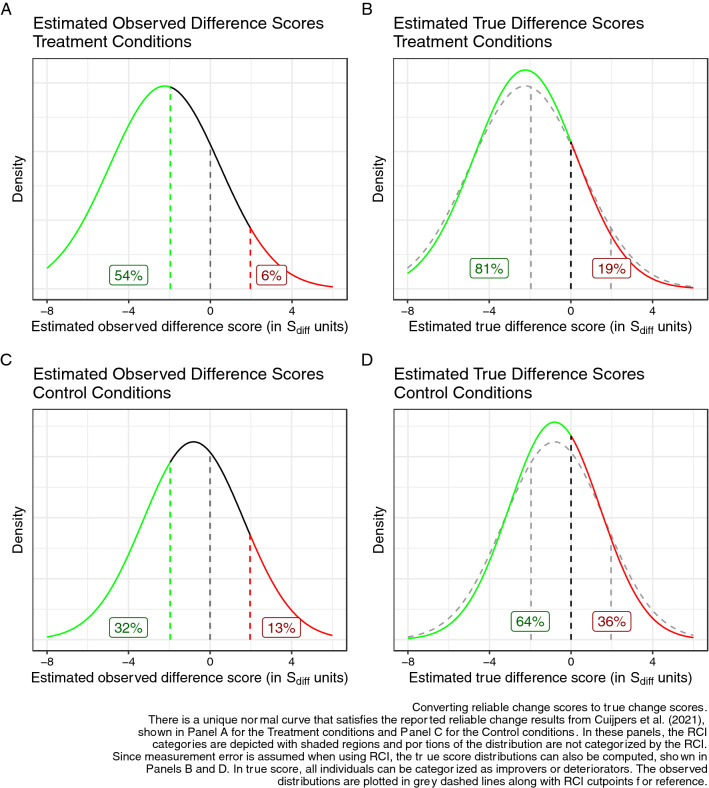
Converting reliable change score distributions to true change score distributions

By doing this, we can see that the true score distribution for the authors’ meta-analytic summary is a normal curve with mean = − 2.23 and SD = 2.50, in units of S_diff_. This process, which again is simply a restatement of the authors’ findings, is depicted in Fig. 1, and reproducible code can be found at https://osf.io/kfnst/.

The fact that these are true scores, thus free of measurement error, makes interpretation much simpler. One of the central problems with the RCI is that it fails to classify large numbers of individuals. In this meta-analysis, for instance, 40% of the treatment sample and 55% of the control sample were unclassified by the RCI. In contrast, there is no reason to avoid classifying individuals on true scores.

As can be seen in Fig. 1 and Table 1, the comparison of true scores makes the deterioration rates even more concerning. The Authors report 6% of the active treatment sample reliably deteriorated, roughly one in 17 patients. However, in true scores, 19% of this sample deteriorated: roughly one in five patients. At the same time, 36% of patients in the control groups, roughly one in three, truly deteriorated. The percent of individuals who improved also increases by this method, though interpreting this is more complex, because individuals who engage a treatment for depression likely expect improvements of substantial magnitudes to justify the costs (financial, emotional, time) of treatment, so not everyone who improves on true score would consider their outcome good. However, it is reasonable to treat any true score deterioration as a serious problem: patients should not become more depressed during treatment. Therefore, the true deterioration rate is better thought of as a lower bound on negative outcomes: it is likely that a larger portion had outcomes that were not satisfactory.

**Table 1:** Reported and implied values from Cuijpers et al. [1] meta-analysis of treatments for depression in youth

	% Reliably improved	% Reliably deteriorated	% True improved	% True deteriorated	Implied Mean of Observed Difference Scores	Implied SD of Observed Difference Scores	Implied Mean of True Difference Scores	Implied SD of True Difference Scores
Psychotherapy	54	6	81	19	− 2.23	2.70	− 2.23	2.50
Controls	32	13	64	36	− 0.81	2.46	− 0.81	2.24

The authors’ interpretation is nevertheless reasonably consistent with this re-analysis. Patients in the active treatments had notably better outcomes than patients in the control conditions, even though many patients in the active treatments retained residual symptoms of depression after care. There remains no reason to consider the treatments ineffective compared to control. However, focusing exclusively on large difference scores, which is what criteria like the RCI and 50% symptom reduction explicitly do, obscures important findings. For instance, the authors write: “It is encouraging that deterioration rates are lower in treatment than in control conditions, but 6% is still a large proportion” (p. 11). If 6% is large, we need different adjectives for the 19% of active psychotherapy participants and 36% of the controls who actually deteriorated. We must recognize that even in well-supported clinical trials, with expert therapists trained using evidence-based treatments, one in five patients becomes more depressed during care.

This is still clearly better than the control conditions, in which one in three patients apparently deteriorate. Considering that other syntheses, including some by the a related authorship team [2], report similar reliable deterioration rates in psychotherapy, it seems likely that similar findings are commonplace, and not specific to the studies, population or treatment conditions in this meta-analysis.

Like the authors’ original analysis, this re-analysis relies on strong assumptions, especially regarding the normality of distributions. Other assumptions required for the RCI, particularly the authors’ assumptions about reliability coefficients, are relatively weakly justified, so the specific findings from this and the original RCI results should be taken with caution. In spite of this, because the only information that has been used was part of the authors’ results, we can have equal confidence in the authors’ RCI results and the true score distributions they imply.

Even though the active psychotherapies offer an unambiguously superior outcomes compared to controls for depressed youth, roughly a fifth of patients become more depressed during these treatments, and the RCI underemphasizes these effects. In future analyses, true-score distributions may be more useful than the RCI, especially for deterioration. Clinicians and researchers should acknowledge these real negative outcomes, and we must strive to further improve the available mental health treatments.
